# Combined Endoscopic-Transcutaneous Approach for Management of Large Parotid Stones

**DOI:** 10.22038/ijorl.2020.43460.2440

**Published:** 2020-11

**Authors:** PP Singh, Megha Goyal, Ankur Batra

**Affiliations:** 1 *Department of Otorhinolaryngology and Head & Neck Surgery, University College of Medical Sciences and Guru Teg Bahadur Hospital, Delhi-110095, India.*

**Keywords:** Combined approach, Parotid stones, Sialolithiasis, Sialendoscopy, Transcutaneous

## Abstract

**Introduction:**

The aim of this paper is to present our experience with combined endoscopic-transcutaneous approach in terms of effectiveness and safety in patients with large or impacted parotid stones.

**Materials and Methods:**

This is a prospective study carried out from August, 2012 to February, 2017 analyzing 21 patients with parotid sialolithiasis. The indication of combined approach was either failed attempt to remove the stone endoscopically, large size (>4mm), or impacted stone. The exact location of the stone was pointed out by endoscopic transillumination and the stone was removed via transcutaneous incision which could be linear incision or a preauricular incision followed by stenting for 3 weeks.

**Results:**

We were successfully able to remove the stone in all 21 cases using modified Blair’s incision in 18 cases, while a linear incision was used in remaining 3 cases. Two patients developed stricture in the post-operative period at 5 and 3 months, respectively. The strictures were successfully dilated endoscopically and the patients are asymptomatic ever since.

**Conclusion:**

Combined endoscopic-transcutaneous approach is a highly successful approach with few complications for removal of parotid stones and thus resulting in high gland preservation rates in patients of parotid sialolithiasis.

## Introduction

Sialolithiasis is the most common cause of obstructive sialadenitis forming 60% of all causes (1,2). Submandibular gland is most commonly affected by sialolithiasis (70-80%) while in about 10-20% of cases, parotid gland is involved (3,4). With the introduction of sialendoscopy, a significant number of the stones can be removed via endoluminal techniques. However, around 5-10% of cases in which either the stone is impacted into the duct wall due to chronic inflammation or the duct is stenotic distal to the stone or the stone is of large size (>5mm); these cases pose surgical challenge as they are not amenable for endoscopic removal (5,6). These cases can either be removed by sialendoscopy guided laser fragmentation or via extracorporeal shock wave lithotripsy (ESWL). The laser beam allows fragmentation of stone and then removal with the basket. ESWL similarly fragments the stone into smaller pieces but may require multiple sessions of lithotripsy (7). Capaccio et al reported 7mm as the upper limit of stone fragmentation by ESWL (8) and in addition, the universal unavailability has restricted the use to a few centres worldwide (5,6). Similarly, the endoluminal laser is not available everywhere and also in unexperienced hands, the laser beam can hit the ductal wall causing perforation and the heat generated in the process may lead to stricture formation in future (9). Even after using ESWL or laser fragmentation, up to 10% of stones can’t be removed. 

For these cases, an alternate combined endoscopic-transcutaneous technique was described by Nahlieli et al in 2002, the exact location of the stone is pointed out by endoscopic transillumination and the stone is removed via transcutaneous incision which could be linear incision or a preauricular incision (10,11). This approach has proven to be highly successful as described by Koch et al, Marchal, Walvekar et al, Capaccio et al, among others (5,12-14). The aim of this paper is to present our experience with this approach in terms of effectiveness and safety in select patients with large or impacted parotid stones.

## Materials and Methods

This is a prospective study carried out in department of Otorhinolaryngology and Head Neck surgery of a tertiary care hospital from 

August, 2012 to February, 2017. During this period, a total of 310 parotid sialendoscopy were done in our department. Out of these, the stone was the cause of sialadenitis in 73 patients. The stone could be removed endoscopically in 52 patients, while 21 patients required combined approach for stone removal. A CT scan was done for all of the patients pre-operatively to accurately map the size, number and site of stone. Out of these, a diagnostic sialendoscopy was done previously in 7 patients under local anesthesia solely on the basis of history. The indication of combined approach was either failed attempt to remove the stone endoscopically, large size (>4mm), or impacted stone.The study was approved by the ethics committee of our institute. A written and informed consent was taken from all the patients explaining the details and complications of the procedure. Patients were given the option of the procedure in either local or general anesthesia. 

*Surgical technique*

**Fig 1:**
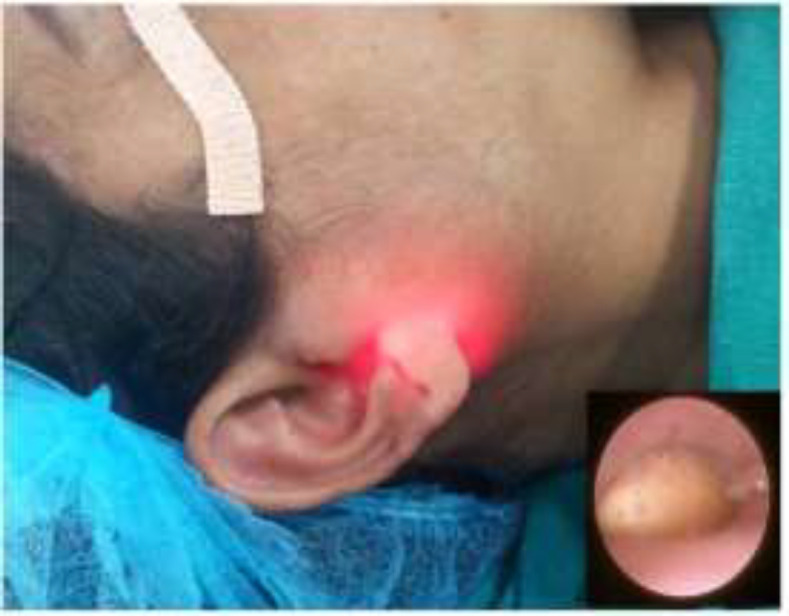
Trans-illumination effect on skin at the location of stone. The inset shows the sialendoscopic view at the same time

In all the patients, the procedure was carried out under general anesthesia. First step of this approach was to perform a sialendoscopy using 0.9mm semi-rigid endoscope (Marchal All-in-one miniature endoscope, Karl Storz, Tuttlingen, Germany) and the stone was visualized in the duct. The sialendoscope was then fixed at the angle of mouth using adhesive tapes and the position of the patient was changed to a classical parotidectomy position. The corresponding site of stone on the cheek skin was marked using trans-illumination of the endoscope as the guide (Fig.1).

**Fig 2:**
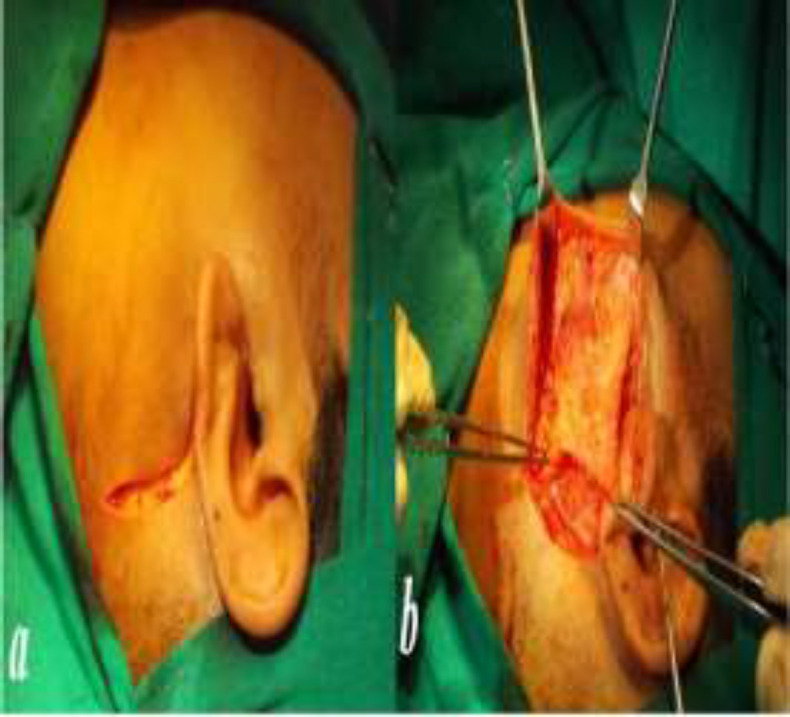
***a*** ***.*** The modified Blair’s incision. ***b.*** Elevation of SMAS flap

**Fig 3:**
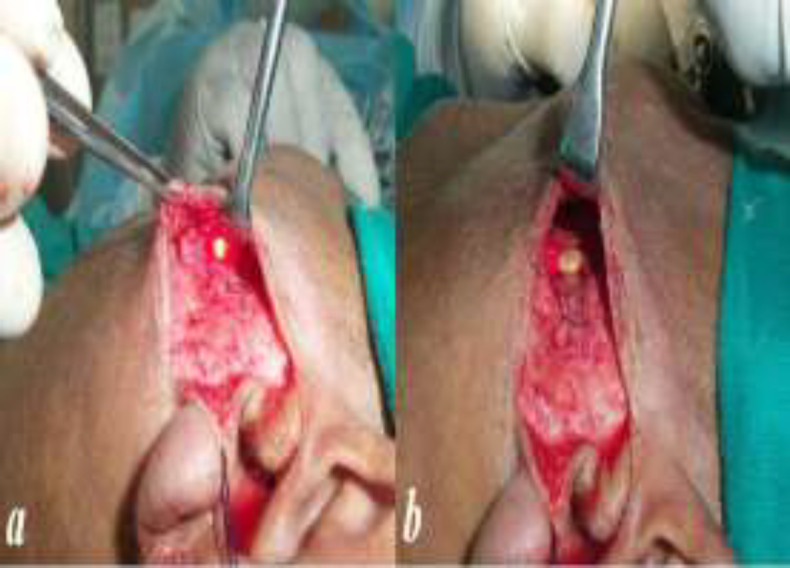
***a. ***Illumination in the duct at the site of stone after the elevation of flap. ***b .***Stone extracted after incising the duct

The illumination was then turned off and could be switched on as and when required intra-operatively. Then the skin incision, either preauricular (modified Blair’s) incision (Fig.2) or a horizontal incision (Fig.3) over the marked area was made. 

In case of proximal ductal or hilar stones, we used a modified Blair’s incision while in case of stones distal to massetric bend, we preferred linear incision. In case of modified Blair’s incision, a superficial musculoaponeurotic system (SMAS) flap was created after raising the skin flap (Fig.2). 

**Fig 4:**
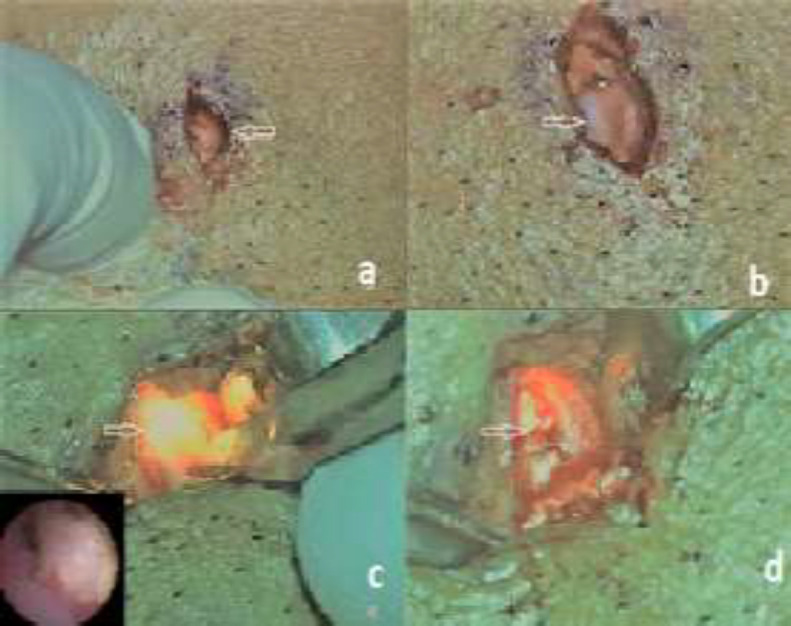
**a. **Horizontal skin incision (arrow).** b.** Impression of ductal stone seen after blunt dissection (arrow). **c.** Illumination in the duct at the site of stone (subset shows simultaneous endoscopic visualization of stone). **d.** stone seen after incision over the duct (arrow).

The duct was isolated with the help of an operating microscope. We tried to visualize and preserve the buccal branch of facial nerve and its divisions as they are in close proximity to the duct. A horizontal incision was then directly placed over the stone and the stone was extracted under direct vision (Fig.4).

The duct was then repeatedly flushed with saline to wash out the debris and a check endoscopy was done by passing the endoscope through the incision site over the duct to visualize the proximal ducts and peripheral branches. The duct was stented with infant feeding tube no. 5 (Romsons International, Noida, India) and closed over the stent with 5-0 absorbable suture. The skin was then closed in layers. The stitches were removed on the 5^th^ day while the stent removed on 21^st^ day. All the patients received antibiotics for five days post-operatively.

*Follow-up*

The patients were followed-up weekly till first month and thereafter every six monthly. The stent was removed at the third visit at 3 weeks post-operatively. During the first 15 days, the patients were asked to massage the gland. During follow-up, the evaluation was done for wound healing, patient symptomatology and patient satisfaction. All the patients currently under follow-up with a minimum of 24 months are included in this study.

## Results

The mean age of the patients in our study group was 36.3 years (min. 6/max. 64) with 10 males and 11 females (Table1). The mean duration of symptoms was 34.15 months ranging from 1 month to 172 months with recurrent swelling and intraoral discharge being the commonly associated symptoms. Of the 21 patients, 7 patients previously underwent diagnostic sialendoscopy and failed attempt at endoscopic stone removal in local anesthesia. Other patients underwent ultrasonography (USG) initially which confirmed the diagnosis of parotid stones. All of the 21 patients had CT scan prior to surgery to accurately know the size, site and number of stones pre-operatively. The mean stone size on CT was 6.4mm. All the procedures were done in general anesthesia. Initially a 0.9 mm sialendoscope was passed to confirm the position of the stone and mark the site for transcutaneous incision. Regarding the position of the stone; proximal to massetric bend in 15 patients, distal in 3 patients, 2 patients had hilar stone while one patient had two stones (one distal and one proximal). 

**Table 1:** Clinical and endoscopic findings, investigations, and details of parotid stones (abbreviations: M/F-male/female; R/L/B-right/left/bilateral; SIAL-diagnostic sialendoscopy, CT-computed tomographic scan, USG-ultrasonography; Y/N-yes/no)

**NUMBER**	**Age** (Years)	**Gender** (M/F)	**Side** (R/L/B)	**Pre-Op Investigation**	**Number**	**Palpable** (Y/N)	**Size** (Mm)	**Site**	**Endoscopyfindings**	**Incision**	**Complications**	**Management**	**Follow-Up**	**Comments**
**1.** **2.** **3.** **4.** **5.** **6.** **7.** **8.** **9.** **10.** **11.** **12.** **13.** **14.** **15.** **16.** **17.** **18.** **19.** **20.** **21.**	52 8 54 24 61 20 6 64 58 32 38 41 26 50 41 40 34 30 34 20 30	M F M F F M F M M F F M M F F F M F M F M	R L L R R R R R R L L R L R R L L L L R L	SIAL+CT SIAL+CT SIAL+CT SIAL+CT SIAL+CT SIAL+CT SIAL+CT USG+CT USG+CT USG+CT USG+CT USG+CT USG+CT USG+CT USG+CT USG+CT USG+CT USG+CT USG+CT USG+CT USG+CT	1 1 1 1 1 1 1 1 1 1 1 1 1 1 1 1 2 1 2 1 1	N N N N Y N N Y N N N N N N N N N N Y N N	4.5 6.2 6.5 7 6.5 5.8 4.8 8.2 7 7 5 5.2 5.5 6.8 5.8 6.5 5.5,4 7 7,3.2 7 6.7	Proximal Proximal Proximal Proximal Distal Proximal Proximal Distal Proximal Distal Proximal Proximal Proximal Hilum Hilum Proximal Proximal Proximal Proximal, Distal Proximal Proximal	stricture distal distal stenotic segment distal stenotic segment papillary stenosis membranous stricture	Blair’s Blair’s Blair’s Blair’s Linear Blair’s Blair’s Linear Blair’s Linear Blair’s Blair’s Blair’s Blair’s Blair’s Blair’s Blair’s Blair’s Blair’s Blair’s Blair’s	Stricture Discharge Stricture	Dilatation Conservative Dilatation	52 52 48 48 46 46 44 42 44 42 40 40 40 36 34 32 31 31 31 28 28	At 5 months At 3 months

The distal stone was removed using a wire basket endoscopically and measured 3.2mm while the proximal stone had to be removed via combined approach. Other endoscopic findings were; stenotic segment of duct distal to the stone in two patient while two other patients had stricture distal to the stone. The stenosis and strictures were dilated endoscopically with serial sizes of endoscopes. In 18 patients, a classical modified Blair’s incision was given and SMAS flap elevated and a linear skin crease incision was used in three patients. All the ducts were stented for 3 weeks.

**Fig 5:**
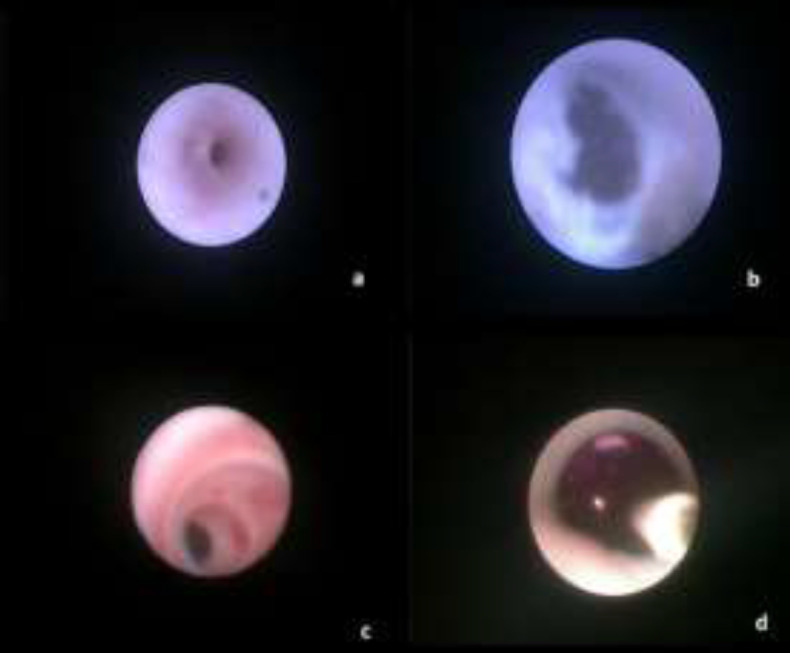
**a, b** ***: ***Pre and post stricture dilatation (Patient 2) **c, d*: ***Stricture pre dilatation and balloon dilatation in progress (Patient 21)

The mean hospital stay was 3 days and all the patients received intravenous antibiotics during this period. One patient had swelling and intraoral purulent discharge 15 days after the procedure. The patient was given oral antibiotics for two weeks coupled with sialoguoges and glandular massage, the sialadenitis resolved without any sequelae and the patient is asymptomatic ever since. Two patients complained of recurrent swelling, 5 and 3 months respectively after the procedure. A diagnostic sialendoscopy was done for both patients in local anesthesia which revealed a stricture in the area of previous ductal incision (Fig.5). The stricture was dilated and the duct stented for 3 weeks. We didn’t encounter other complications such as salivary fistula or facial nerve paresis (buccal branch). All the patients in our study were satisfied with the scar and none of them developed a hypertrophic scar or keloid. None of our patient needed duct ligation or parotidectomy. The mean follow-up is 39.7 months (min. 28/max. 52). 

## Discussion

For decades, the standard treatment of parotid stones has been superficial parotidectomy until the advent of sialendoscopy and other minimally invasive techniques (ESWL, laser lithotripsy). For small stones (<4mm, floating), sialendoscopy has become the standard procedure for stone extraction and has significantly increased the gland preservation rates. However, it is large (>4mm) or impacted parotid stones that pose technical challenge and limit the possibility of using sialendoscopy. One of the option is to fragment the large stone into smaller fragments which can be achieved either through ESWL or endoluminal laser fragmentation. The reported success rate in complete elimination with ESWL is around 60% while in another 30%, the symptoms only partially improved. In part of these patients, ESWL can be combined with sialendoscopic removal after fragmentation (4,15,16). Despite sialendoscopy and ESWL, approximately 10 % of sialoliths cannot be removed endoscopically and will continue to be the cause of recurrent inflammations and swellings of the gland. Another choice is to use laser (thulium: YAG) for stone fragmentation with a reported success rate of 80% in the literature. The main risks with laser fragmentation are duct perforation (12.7%) and thermal injury to the surrounding nerves, vessels, and soft tissue. However, both these techniques require expensive devices and this may explain the restricted clinical availability universally (9). The described combined approach is an alternative option in case the above mentioned techniques are not available or have previously failed.

Since the description of the technique by Nahlieli et al in 2002, the technique is slowly gaining popularity and is now indicated in select group of parotid stones (10). According to Nahlieli, the indications for the combined approach were; calculus in the posterior third of the Stensen’s duct with too narrow duct anterior to it, obstruction of the posterior or middle third of the Stensen’s ducts leading to the calculus, large (>5-mm) stones in the middle or posterior part of the duct that cannot be dilated for intraductal removal, and intraparenchymal stones. They used 1cm facial line incision. Of the 12 patients they treated with this approach, 9 had complete removal (75%); in 1 case with 3 sialoliths, they were able to remove 2 and the gland remained asymptomatic. In three patients, they were unable to extract the stones. In 7 cases, the glands returned to function, 3 glands became atrophic with no function, but the gland remained asymptomatic. The aesthetic results were satisfactory in all cases, and no major complications were noted. McGurk et al in 2005 described a similar technique but with preauricular incision (11). They treated eight patients (7 stone and 1 stricture) and were able to successfully remove the stone in seven. In one patient of stone and other with stricture, the duct could not be repaired and had to be ligated. No major complications were noted by them. Marchal in 2007 reported similar results using combined approach (12). In one case with mixed pathology (stone+stricture), the duct restenosed despite dilatation and stenting and finally the duct was ligated. Walvekar et al used the double approach procedure in 19 out of 106 patients with sialolithiasis (18 %). Stones were removed in 90 % of the cases without any complication (13). Koch et al. described his experience with this technique in 19 patients, 17 of which suffered from lithiasis. The treatment was successful in 89.5% of all cases and in 94.1% of the patients with lithiasis, respectively. They had to perform parotidectomy in two cases as it was not possible to reconstruct the duct (17).

According to Capaccio et al, the upper limit of stones that can be removed successfully by ESWL in majority of cases is 7mm (18). For large stones (>7mm), they used a sialendoscopy-assisted transfacial surgical approach that was effective in all (7 out of 8) but one case, which was successfully solved by combining this procedure with extra-corporeal lithotripsy and operative sialendoscopy (14). In our group, we were able to successfully remove the stones (21/21) in all of the patients without the need of parotidectomy or Stensen’s duct ligation in any of the patients.

The placement of stent after the procedure is still a matter of debate, although stenting the duct is essential to prevent stenosis, fistulas or sialoceles (19). On the contrary, Numminen et al report successful combined treatment in 6 of the 8 patients without the use of stent (20). Inspite of stenting in 9 out of 12 patients, Konstantinidis et al reported mild ductal stenosis on postoperative endoscopic evaluation but without clinical significance as no recurrent swellings were reported (21).

We used infant feeding tube as stent in all of the patients without any issue. However, two of our patient complained of recurrent swelling and discharge at 3 and 5 months post-operatively. 

A diagnostic sialendoscopy was done which revealed stricture in the area of ductal incision which was endoscopically dilated and the duct restented for 3 weeks. Both the patients have been asymptomatic ever since. 

No other major or minor complications were noted. We didn’t encounter any sialoceles or salivary fistulas in our patients, although it has been reported that such complications affected 10% of the patients in McGurk's series (though it was temporary) (6). None of the patient in our series developed temporary or permanent facial palsy. Cosmetically, all the patients were satisfied with the scar. None of them developed a hyertrophic scar. The type of skin incision in our study depends on the location of the stone. For stones proximal to masseteric bend, we used preauricular incision while a horizontal linear incision was used for stones located in the distal duct (19). In our group, we used a preauricular incision in 18 patients while linear skin crease incision was used in 3 patients. The procedure can be performed either under general or local anesthesia depending on the location of stone and patients preference. In our study, all the procedures were done under general anesthesia. The post-operative results were good and satisfactory both for the patients and the surgeon. 

## Conclusion

Combined endoscopic- transcutaneous approach is a highly successful approach with very few major complications for removal of parotid stones and thus resulting in high gland preservation rates in patients of parotid sialolithiasis. The indications of combined approach includes: large parotid stones (>4mm), impacted stones, or failed attempt at endoscopic removal. In the absence of ESWL or laser lithotripsy, this approach can be recommended for the abovementioned indications without significant morbidity.
